# Evaluation and Comparison of Mechanical Properties of Polymer-Modified Asphalt Mixtures

**DOI:** 10.3390/polym13142282

**Published:** 2021-07-12

**Authors:** Hamad Abdullah Alsolieman, Ali Mohammed Babalghaith, Zubair Ahmed Memon, Abdulrahman Saleh Al-Suhaibani, Abdalrhman Milad

**Affiliations:** 1 Department of Civil Engineering, College of Engineering, King Saud University (KSU), Riyadh 11451, Saudi Arabia; halsolieman@ksu.edu.sa (H.A.A.); Asuhaib@ksu.edu.sa (A.S.A.-S.); 2 Center for Transportation Research, Department of Civil Engineering, University of Malaya (UM), Kuala Lumpur 50603, Malaysia; 3 Department of Engineering Management, College of Engineering, Prince Sultan University (PSU), Riyadh 12435, Saudi Arabia; 4 Department of Civil Engineering, University Kebangsaan Malaysia (UKM), Selangor 43600, Malaysia; miladabdalrhman@siswa.ukm.edu.my

**Keywords:** polymer-modified asphalt, mechanical properties, dynamic modulus, Hamburg wheel-tracking, indirect tensile strength

## Abstract

Polymer modification is extensively used in the Kingdom of Saudi Arabia (KSA) because the available asphalt cement does not satisfy the high-temperature requirements. It was widely used in KSA for more than two decades, and there is little information regarding the differences in the performance of different polymers approved for binder modification. Pavement engineers require performance comparisons among various polymers to select the best polymer for modification rather than make their selection based on satisfying binder specifications. Furthermore, the mechanical properties can help select polymer type, producing mixes of better resistance to specific pavement distresses. The study objective was to compare the mechanical properties of the various polymer-modified asphalt (PMA) mixtures that are widely used in the Riyadh region. Control mix and five other mixes with different polymers (Lucolast 7010, Anglomak 2144, Pavflex140, SBS KTR 401, and EE-2) were prepared. PMA mixtures were evaluated through different mechanical tests, including dynamic modulus, flow number, Hamburg wheel tracking, and indirect tensile strength. The results show an improvement in mechanical properties for all PMA mixtures relative to the control mixture. Based on the overall comparison, the asphalt mixture with polymer Anglomk2144 was ranked the best performing mixture, followed by Paveflex140 and EE-2.

## 1. Introduction

Saudi Arabia roadways had evolved highly through the previous decades. Flexible pavement is the dominant type used for these roads. The increase in heavy truck loads had led to premature rutting in the asphalt layer of roadway pavements. To control the asphalt pavement deformation, it was suggested to use stiffer asphalt binder to sustain the heavy truck loads. Implementation of SUPERPAVE specifications showed that the available asphalt binder was not hard enough at high service temperature. Asphalt binders can be made harder by modification. Different types of modifiers were tried worldwide to enhance the rheological properties of asphalt binder, such as polymers [1,2,3,4,5,6], crumb rubber [5,7,8,9,10,11], waste plastic [12,13,14,15,16], geopolymers [17], and nano-materials [18,19,20,21]. Polymers are commonly used to modify and improve the rheological properties of asphalt binders. Polymer modification of asphalt binders for pavement construction can increase its resistance to permanent deformation at high temperatures and its resistance to thermal cracking at low temperatures. These possible improvements can increase pavement life [22,23,24,25,26]. There are two main kinds of polymers—namely, elastomers and plastomers. The elastomers are usually used to extend the binder’s low and high service temperatures. However, plastomers are notable as effective additives that can raise the high service temperature [12,27,28]. The polymer-modified asphalt (PMA) properties depend upon two parameters: the first one is the material properties such as polymer type, polymer content, asphalt binder grade, and asphalt source [29]; the second is the mixing process of asphalt binder and modifiers [5,30,31]. Several studies have explored the effect of using modifiers on asphalt binder and mixture properties. The results of these studies showed that polymer modification could alter binder properties by increasing the softening point [1,2,3,4,14,32,33,34,35,36,37,38], increasing the viscosity [3,37,39,40], decreasing the penetration [2,3,14,37,38], and improving the performance grade [2,4,6,37,41]. For asphalt mixture, the previous studies indicated that polymers could improve the mechanical properties of asphalt mixture, such as resilient modulus [14,42,43,44,45,46], fatigue cracking resistance [4,14,47,48,49,50,51], and rutting resistance [43,50,52,53,54,55,56].

**Figure 1:**
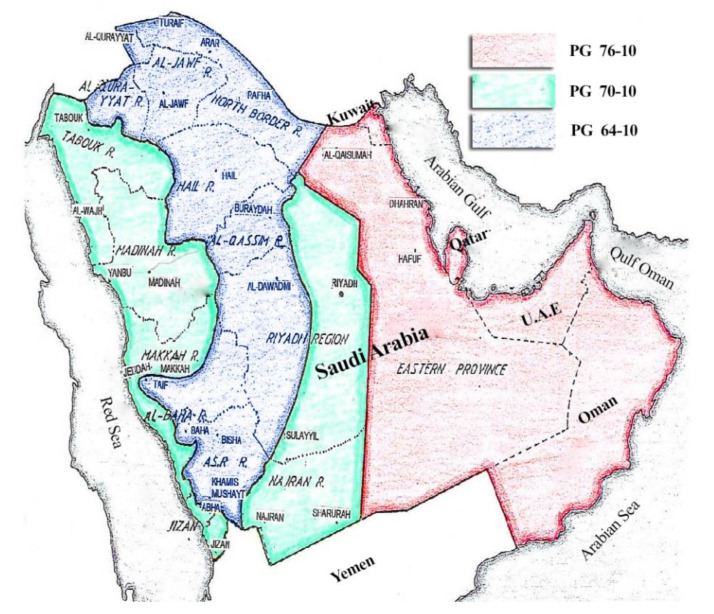
The temperature zoning for Gulf countries.

The asphalt binder produced in the Kingdom of Saudi Arabia (KSA) is only one penetration grade, 60–70, which satisfies the performance grade (PG) specification PG64-22 [57]. Field measurement of pavement temperatures in KSA revealed that the asphalt pavement temperature ranges between 3 °C and 72 °C for coastal areas, and between 4 °C and 65 °C for inland areas [58,59]. Therefore, the recommended high-temperature grade of asphalt binder for Riyadh city is higher than 64 °C by one grade, as presented in Figure 1.

This available asphalt binder grade (PG64-22) is not satisfactory for Riyadh and other regions of KSA where high-temperature conditions prevail. It is not satisfactory for high-traffic roads and slow-speed and stationary conditions such as road intersections. Therefore, the asphalt binder needs to be modified to meet the requirements of local climate and traffic conditions. To overcome premature pavement distresses, the Ministry of Transportation (MOT) and Riyadh Municipality (RM) implemented SUPERPAVE™ mix design, which improved materials selection and mixed design procedures. Implementation of SUPERPAVE™ specification increased the demand for the utilization of polymer for asphalt modification. As a result, many asphalt plants produce modified asphalt binders to satisfy the performance grade specification. Many types of polymers were approved by the MOT for pavement construction. Although polymer-modified asphalt was widely used in KSA for more than two decades, there is little information regarding the differences in performance of different types of polymers approved by the MOT for binder modification. Pavement engineers require performance comparisons among the various polymers to select the best polymer for modification rather than making their selection based on satisfying binder specifications. Therefore, there is a need to investigate the properties of the various PMA produced by asphalt plants in the Riyadh region and to extend the evaluation to the mechanical properties of their asphalt mixtures. These properties can help select polymer types that produce mixes of better resistance to specific pavement distresses. The main objective of this study was to evaluate and compare the mechanical properties of various PMA mixtures which are widely used in the Riyadh region, as well as to compare the results with a range of mixtures containing the original binder (un-modified). Dynamic modulus, flow number, Hamburg wheel tracking, and indirect tensile strength tests were conducted on the control mix and five other mixes prepared with different PMA (Lucolast 7010, Anglomak 2144, Pavflex140, SBS KTR 401, and EE-2).

## 2. Materials

### 2.1. Asphalt Binder

Asphalt cement produced in KSA has a performance grade PG64-22 (60/70 penetration grade). Table 1 presents the properties of the asphalt binder.

### 2.2. Aggregate

**Figure 2:**
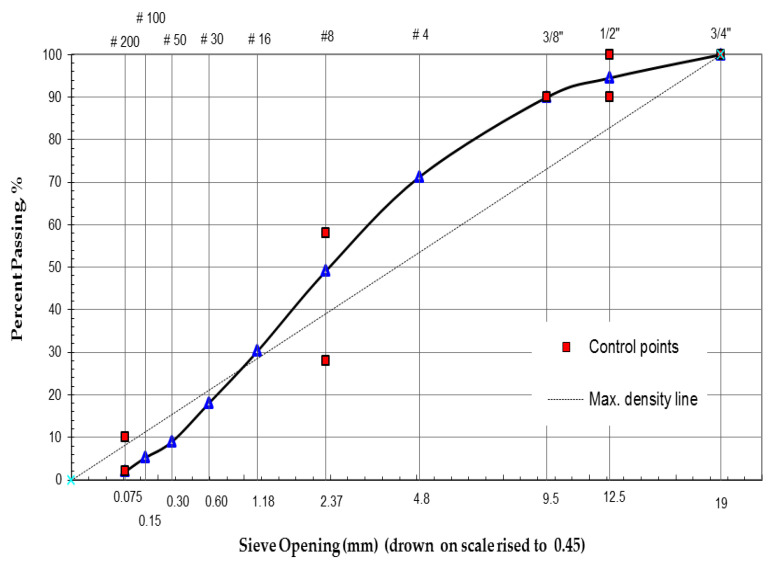
Aggregate gradation.

In this study, the aggregate used was limestone procured from a hot mix plant located near Riyadh city in Saudi Arabia. In order to ensure precise gradation, the aggregate was sieved into several sizes and combined to get the specified gradation that would satisfy the maximum and minimum limits of aggregate percentage passing according to the Ministry of Transportation of KSA specification [60]. The aggregate gradation used in this study was dense-graded, as shown in Figure 2 and Table 2. The physical properties of limestone aggregate are presented in Table 3.

### 2.3. Polymer-Modified Asphalt

**Figure 3:**
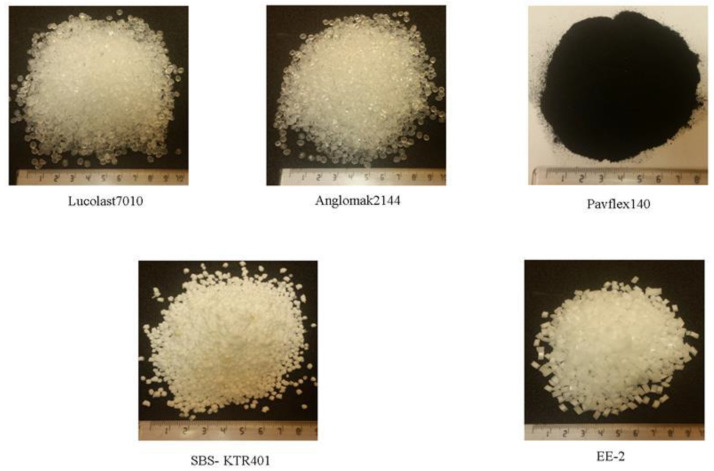
Types of modifiers evaluated.

Five types of polymers were selected, which represent polymers widely used in the Riyadh region. These polymers were Lucolast 7010, Anglomak 2144, Pavflex 140, SBS KTR 401, and EE-2. All polymers used in this study were in pellet and powder form, as shown in Figure 3. The physical and chemical properties of those modifiers are tabulated in Table 4. The base asphalt binder was mixed with the specified polymer using an asphalt blender. The polymer content for each polymer was determined so that it reached the required PG 76-10 set by the KSA Ministry of Transportation, as shown in Table 5. As mentioned before, polymer modification needed to satisfy the high-temperature grade of 76 °C, which is required for the Riyadh region and other hot regions of KSA [61].

## 3. Mix Design and Experimental Program

HMA was prepared according to SUPERPAVE Volumetric Mixture design (AASHTO PP28-95) “Standard Practice for SUPERPAVE Volumetric Design for HMA” and KSA Ministry of Transportation specification for asphalt mixture design [60]. To optimize the binder content, three duplicate samples were prepared at four different contents of asphalt binder: 4.5, 5.0, 5.5, and 6.0% (by total weight of mixture). For each sample, the aggregate was merged with an asphalt binder at 155 °C then placed in the oven at 135 °C for 2 h to cure. Then the specimens were moved into another oven at 145 °C for half an hour and compacted by a SUPERPAVE gyratory compactor using a design number of gyration (Ndes) equal to 100 gyrations. Another set of specimens was also mixed and left loose to determine maximum theoretical specific gravity (AASHTO T209). The bulk specific gravity of each compacted specimen was measured according to AASHTO T166 test method and was used to calculate the volumetric parameters (AV, VMA, and VFA) according to AASHTO PP 19. The average volumetric properties for the control mix are summarized in Table 6.

The optimum asphalt content was defined as the percentage that produced 4.0% air void. At 4.0% air void, an asphalt mixture will show less asphalt bleeding and better rut resistance [62]. The optimum asphalt content was found equal to 5.20% by the total mixture weight and satisfied all the mix requirements according to the specifications of the Ministry of Transportation [60]. For polymer-modified asphalt mixtures, it was decided to use the same aggregate structure and optimum binder content (5.2%) obtained for the control asphalt mixture. This was to make comparing the characteristics of mechanical asphalt mixtures easier without having to take into account other factors such as aggregate structure and binder content. However, the mixing and compaction temperatures were increased to 165 °C and 155 °C, respectively, to take into consideration the increase of binder viscosity due to modification. Table 7 summarizes the volumetric parameters for mixtures corresponding to 5.2% binder content.

## 4. Mechanical Properties Tests

The designed mixtures were subjected to different performance tests. They are described in the following sub-sections. 

### 4.1. Dynamic Modulus (|E*|) Test

**Figure 4:**
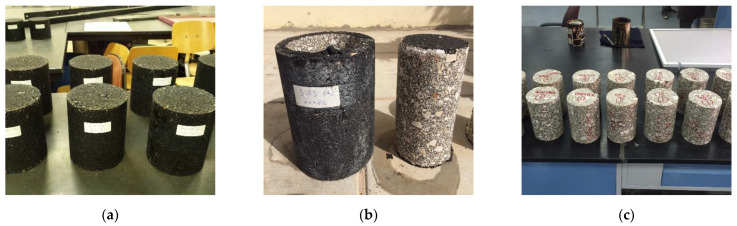
Preparation of test specimens (**a**) compacted specimens, (**b**) Cored specimens, (**c**) ends cut specimens.

The test was used to obtain asphalt mix stiffness. It was performed according to AASHTO TP 62-07 using an asphalt mixture performance tester (AMPT). The test was performed according to AASHTO TP 62-07. The stress levels were varied with the frequency to keep the specimen response within linear viscoelastic limits (recoverable micro-strain below 150 microstrains). The test parameters, dynamic modulus, and phase angle (δ) were measured at four temperatures; −10, 4.4, 21.1, and 54.4 °C and frequencies: 25, 10, 5, 1, 0.5, and 0.1 Hz. The specimens were compacted with dimensions of 15 cm diameter and 17 cm tall using the SUPERPAVE gyratory compactor. First, the samples were compacted to target air voids of 7%. Consequently, the samples were cored from the center to 10 cm diameter and cut from the top and bottom to get the height of 15 cm as shown in Figure 4.

### 4.2. Flow Number (Fn)

**Figure 5:**
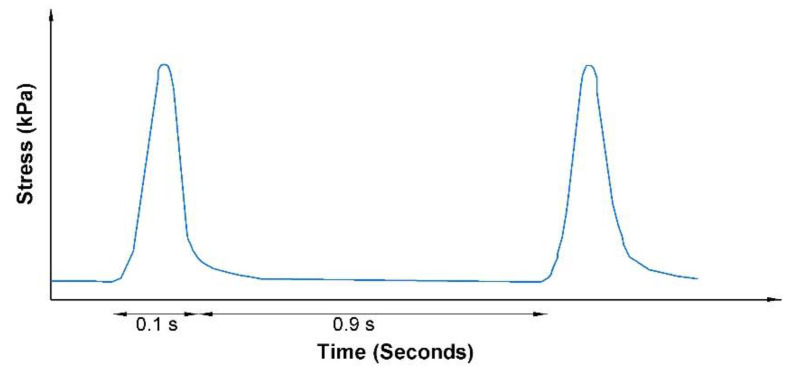
Loading form for Fn test.

Permanent deformation characteristics of HMA mixtures under repeated loading can be determined by using the Fn test. Fn is defined as the number of load cycles corresponding to the minimum rate of change of permanent axial strain during a repeated load [63]. A high Fn value indicates better rutting resistance. The Fn test was conducted using the asphalt mixture performance tester (AMPT) according to the test method described in NCHRP Report 513 [64]. The cylindrical asphalt specimens were subjected to several thousand loading cycles, and the cumulative permanent deformations were recorded as a function of loading cycles. The load was a repetitive vertical axial stress of 600 kPa for 0.1 s, followed by a rest period of 0.9 s, as shown in Figure 5. The test was conducted at a temperature of 76 °C, equal to the pavement’s high service temperature. The failure criterion of this test was either 10,000 cycles or 50,000 microstrains, either of which was first reached. There are three phases to the cumulative permanent strain curve: primary phase, secondary phase, and tertiary phase. The Fn specifies the starting point or cycle number at which the tertiary phase begins. Specimens for this test were prepared in the same way as those prepared for the dynamic modulus test.

### 4.3. Hamburg Wheel Tracking (HWT) Test

**Figure 6:**
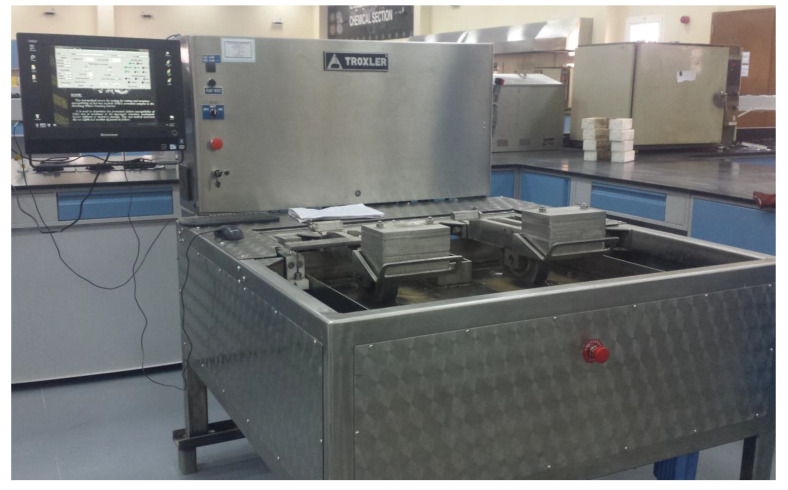
The Hamburg wheel tracker.

The test was performed according to AASHTO T 324 using a Hamburg wheel-tracker. The test was intended to determine how vulnerable HMA was to failure due to defects in the aggregate structure, a lack of binder coating, and poor binder–aggregate adhesion. As shown in Figure 6, the HWT tester is an electrically powered device-driven apparatus that has a rotating steel wheel with a diameter of 203.6 mm and a width of 47 mm. The wheel applies a force of 7054.5 N. The wheel reciprocates over the mid-span of the specimens at a rate of 52 ± 2 pass/min across the specimen.

**Figure 7:**
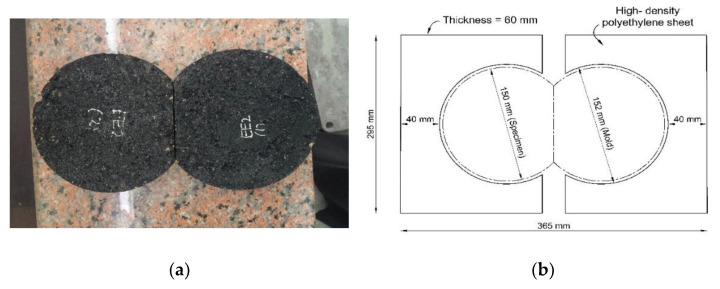
(**a**) Cut edge samples, (**b**) high-density polyethene mold.

The specimens of each mix design were formed with 150 mm diameter 62 ± 2 mm thickness gyratory compacted specimens. Specimens were cut vertically at the edge to be placed back-to-back in a high-density polyethene mold, as shown in Figure 7. The specimens were conditioned in water at a temperature of 50 ± 1 °C with 60 min of water temperature stabilization using a mechanical circulation system. The specimens’ rut depth and the number of passes were recorded. The test ended when the rut depth reached 12.0 mm or 20,000 passes, whichever came first.

### 4.4. Indirect Tensile Strength (ITS)

An ITS test was conducted to determine the tensile strength of neat and polymer-modified asphalt mixtures according to AASHTO-T283 using an indirect tensile compression tester. The test was also conducted on wet conditioned samples to determine how sensitive the mixture was to moisture damage. Six specimens were fabricated for each mixture: three in dry condition and three in wet condition. The wet conditioning was performed by submerging samples in a water bath at a temperature of 60 ± 1 °C for 24 h and then at ambient temperature (25 ± 0.5 °C) for 2 h. Following that, a constant deformation rate of 50 mm/min is applied in the diametral direction of the specimen. To determine the tensile strength, the load at failure was recorded, as shown below. The load at failure was recorded and used to calculate the tensile strength as follows:

(1) $$S_{t}=\frac{2P}{\pi \times T\times D}$$

where *St* is the tensile strength (MPa), *P* is the maximum load (N), *T* is the sample thickness (mm), *D* is the sample diameter (mm).

Finally, the tensile strength ratio (*TSR*) was determined using the following equation:

(2) $$TSR=100*\frac{\mathrm{Tensile}\;\mathrm{strength}\;\mathrm{of}\;\mathrm{wet}\;\mathrm{condition}\;}{\mathrm{Tensile}\;\mathrm{strength}\;\mathrm{of}\;\mathrm{dry}\;\mathrm{condition}\;}$$

A higher *TSR* value indicates that the asphalt mix will have better resistance to moisture damage. The *TSR* must be greater than 80% as recommended by AASHTO T 283 and the Ministry of Transportation.

### 4.5. Comparison and Overall Ranking of PMA Mixture Performance

#### 4.5.1. Pair Comparison

To compare the different mixtures pair, the “effect size method” was implemented in this research instead of statistical tests for significance (*t*-test and ANOVA), which were not applicable due to the limited number of data points for the experimental results. Therefore, the results of the statistical test might be misleading [65]. However, based on the difference in the means of the two groups and the standard deviation, the effect size value (*d*) can be determined by the following equation:

(3) $$d=\frac{\left|\bar{x_{t}}\;-\;\;\bar{x_{r}}\right|}{\sqrt{\frac{\left(n_{t}-1\right)s_{t^{2}}+\left(n_{r}-1\right)s_{r^{2}}}{\left(n_{t}+n_{r}\right)}}}$$

where $\bar{x_{t}}$ is the mean of treatment group, $\bar{x_{r}}$ is the mean of the reference group, $n_{t}$ is the number of samples in the treatment group, $n_{r}$ is the number of samples in the reference group, $s_{t}$ is the standard deviation of the treatment group, $s_{r}$ is the standard deviation of the reference group.

#### 4.5.2. Overall Ranking

In order to decide which mix design had better performance, all different mixes were ranked based on a 6-point scale. This could help select the best mix design by each of the asphalt mixture performances, where the mixture with the best performance would be ranked 1 and the mixture with the least (worst) performance would have the highest number. Based on the asphalt mixture performances for the selected asphalt mixtures analyzed in this study, the relative significance of each mix design’s overall rank can be determined using the Relative Importance Index (*RII*) method. The *RII* is computed as:

(4) $$RII=\sum^{\;}\frac{1+A-W}{A*N}$$

where *A* is the highest weight = 6; *W* is the weight given to each performance test and ranges from 1 to 6; and *N* the total number of performance tests.

## 5. Results and Discussions

### 5.1. Dynamic Modulus Result

**Figure 8:**
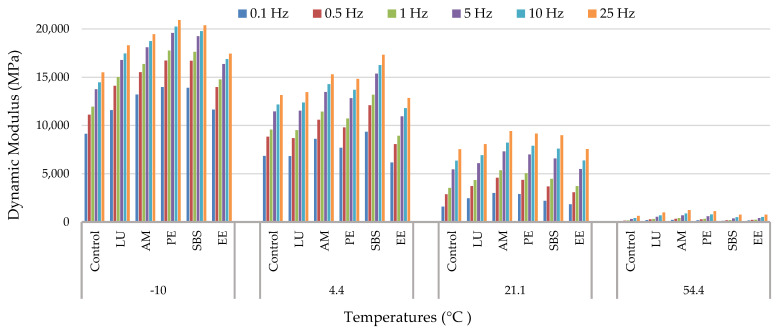
Dynamic modulus versus frequencies at different temperature.

**Figure 9:**
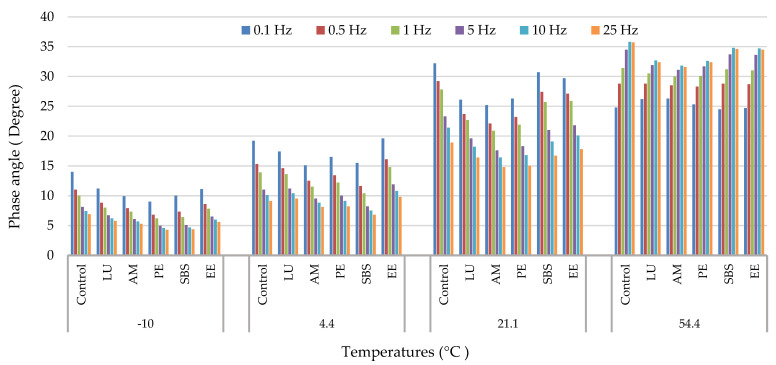
Phase angle versus frequencies at different temperatures.

The experimental data of dynamic modulus (|E*|) and phase angle (δ) versus frequency at different temperatures for different modified asphalt mixtures are presented in Figure 8 and Figure 9, respectively.

Generally, the dynamic moduli values of all modified asphalt mixtures increased by decreasing the temperature, and they were increased by increasing the frequency. While phase angle increased by increasing the temperature, it was decreased by increasing the frequency. This is because as the temperature increases or decreases, the viscosity of the asphalt binder changes, which in turn causes a change in the elasticity of asphalt mixtures. In addition, it is also found that all asphalt mixtures showed similar trends regardless of modifier types. According to many studies [27,42,43,46,66,67,68,69,70], polymer modification resulted in a higher modulus for the modified asphalt mixture as compared with the control asphalt mixture. In this study, similar behavior was found by using different polymers, where the dynamic modulus of asphalt mixtures improved due to polymer addition.

Based on the difference in the means of the two groups and the standard deviation, the effect size values (d) were calculated for different asphalt mixture performance tests, as shown in Table 8, Table 9, Table 10 and Table 11. Based on the literature, an effect size of 1.6 was used in this study to determine the effect of differences in dynamic modulus values of asphalt mixtures on the performance properties [65]. Effect sizes with values less than 1.6 indicate no difference in dynamic modulus values of the two asphalt mixtures. Table 8 presents the effect size values at the temperature of −10 °C; the results show that the Lucolast mixture had statistically no difference (0.26) in dynamic modulus compared with the EE-2 mixture. Additionally, the Paveflex mixture had statistically no difference (0.57) compared with the SBS mixture. 

Table 9 shows the effect sizes for the dynamic modulus of different mixtures at 4.4 °C. It shows that the differences are statistically significant between all asphalt mixtures since the effect size values obtained were greater than 1.6 except for the mixture with Lucolast corresponding to the control mixture.

For a temperature of 21 °C, the results of which are tabulated in Table 10, the control mixture had statistically no difference (0.85) in dynamic modulus compared with the EE-2 mixture. Additionally, the Lucolast mixture had statistically no difference (1.01) compared with the SBS mixture.

Table 11 provides the effect size values at temperature of 54.4 °C, where only the mixture with Lucolast had no difference in dynamic modulus compared with the Paveflex mixture since the effect size values obtained were less than 1.6.

### 5.2. Flow Number (Fn) Result

**Figure 10:**
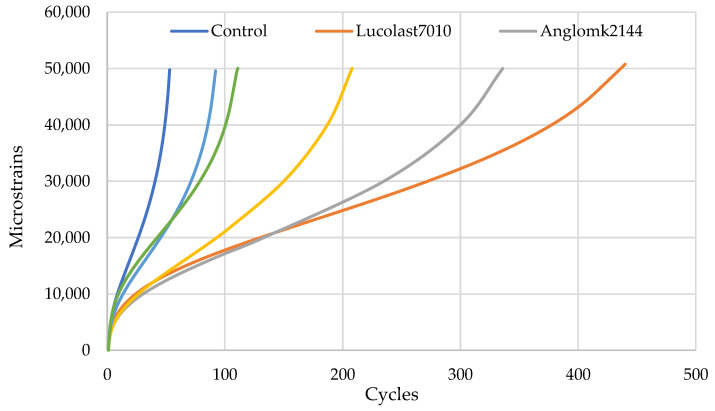
Cumulative permanent strain curves for different mixtures.

Based on the test findings, all asphalt mixtures reached the failure stage with a cumulative permanent strain of 50,000 microstrains. Figure 10 illustrates the cumulative permanent strain curves of different asphalt mixtures. A significant variance was noticed between control and all modified asphalt mixtures. Thus, all mixtures with PMA demonstrated lower permanent strain than the control mixture. This is attributed to the presence of polymer material in the asphalt binder, which can increase the adherence of mixture components, resulting in increased mixture strength.

The Fn and final load cycle of asphalt mixtures are presented in Table 12. Asphalt mixture modified with Lucolast7010 displayed a higher Fn value (182) and reached the failure stage after 432 cycles, followed by the mixture containing Anglomk2144, which showed Fn 120 and reached the failure stage after 336 cycles.

Table 13 provides the effect sizes for the Fn test of different mixtures. It shows that the differences in Fn values are statistically significant between all asphalt mixtures since the effect size values obtained are greater than 1.6.

### 5.3. Hamburg Wheel Tracking Result

**Figure 11:**
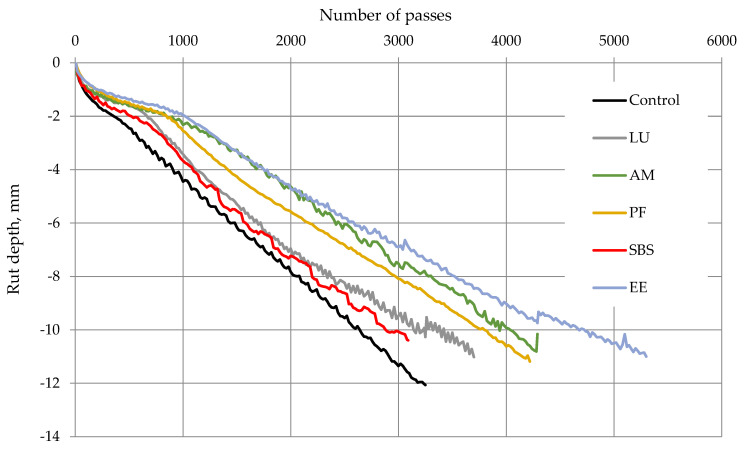
Rut depth versus the number of passes for different mixtures.

The test was used to evaluate rutting and to determine the failure susceptibility because of weak adhesion between the binder and aggregates. Before testing, the specimens were submerged underwater for 60 min at a temperature of 50 °C. All specimens were tested at 52 pass/minute. The specimen’s rut depth and the number of passes were recorded. Testing ended when the rut depth reached 12.0 mm or 20,000 passes, whichever came first. Figure 11 presents the average rut depth recorded with the number of passes for all the mixtures. It is observed that the PMA mixtures had lower moisture sustainability than the neat asphalt mixture. From the figure, the asphalt mix modified with EE-2 ranked as the best mixture, followed by Anglomak2144, Paveflax140, Lucolast7010, and SBS KTR401.

### 5.4. Indirect Tensile Strength Result

**Figure 12:**
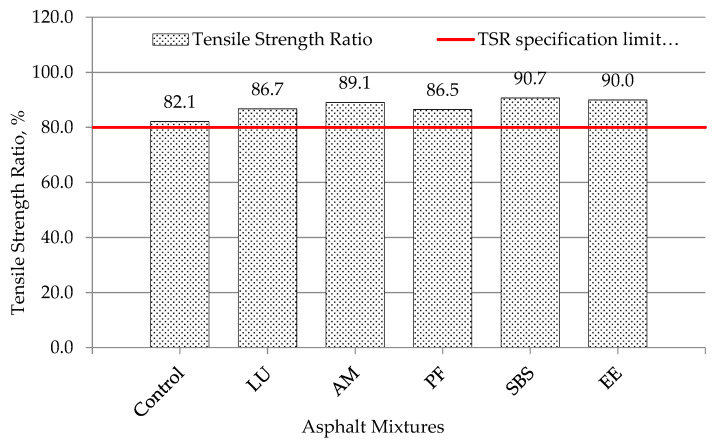
Tensile strength ratio for different mixtures.

This test was conducted to determine the tensile strength and water susceptibility of neat and PMA mixtures using indirect tensile strength tests. The indirect tensile strength values for three specimens in dry and wet conditions of neat and PMA mixes are presented in Table 14. Asphalt mixture modified by SBS KTR401 showed the highest dry strength, while the mixture modified by polymer EE-2 showed the lowest strength compared with other PMA mixtures. The ratio of tensile strength of wet sample to dry sample was determined using Equation 2 and is presented in Figure 12. The results indicate that there were improvements in water susceptibility of polymer-modified mixtures over that of the neat mixture. It is worth mentioning that the tensile strength ratio (*TSR*) values of neat and PMA mixtures were higher than the recommended minimum limit based on SUPERPAVE specification (80%).

Table 15 shows the effect sizes for the *TSR* of different mixtures. It shows that the differences in Fn values were not statistically significant between some asphalt mixtures since the effect size values obtained were less than 1.6. For example, the Lucolast mixture had statistically no difference in *TSR* compared with the Paveflex140 mixture since the effect size value was 0.12.

### 5.5. Overall Ranking of PMA Mixture Performance

The mixes were ranked based on a 6-point scale, where the mixture with the best performance would be ranked as 1 and the mixture with the worst performance would have the highest number, so the worst performance would be ranked as 6, as shown in Table 16. The Relative Importance Index (*RII*) (Equation 4) was used to calculate the mix design’s relative significance for different performance tests. Based on the *RII* values, the overall ranking of asphalt mixture performance was determined. The findings show that asphalt mixture modified by Anglomk2144 was ranked as the best performance mixture (*RII* = 0.722), followed by asphalt mixtures modified by Paveflex140, EE-2, Lucolast7010, and SBS KTR40 (*RII* = 0.630, 0.630, 0.593, and 0.574, respectively).

## 6. Conclusions

In this study, the aim was to evaluate and compare the mechanical properties of the various polymer-modified asphalt (PMA) mixtures. Based on the results and analysis, the following conclusions are offered:

- The dynamic moduli values of all modified asphalt mixtures increased by decreasing the temperature and increased by increasing the frequency. Polymer-modified asphalt mixtures showed higher dynamic modulus values than neat asphalt mixture values for different frequencies and temperatures.
- Modified mixtures showed significant improvement in flow number compared with neat asphalt mixture. Asphalt modified with Anglomak2144, Pavflex140, and Lucolast polymers ranked as the best mixtures to rut resistance.
- Hamburg wheel tracking test results showed that asphalt mixture modified with polymers has better adhesion between the binder and aggregates compared with the neat asphalt mixture. The asphalt mixture modified with EE-2 ranked as the best, followed by Anglomak2144, Paveflax140, Lucolast7010, and SBS KTR401.
- The mixture modified by SBS KTR401 showed the highest indirect tensile strength, while the mixture modified by polymer EE-2 showed the lowest strength compared with other PMA mixtures for dry conditions. For wet conditions, the highest wsa SBS KTR401 and the lowest was Lucolast7010. Moreover, there was an improvement in water susceptibility of PMA mixtures over that of neat asphalt mixture. The tensile strength ratios (*TSR*s) of neat and PMA mixtures were all higher than the recommended minimum value (80%).
- Based on the overall ranking of mechanical properties, the asphalt mixture with polymer Anglomk2144 was ranked as the best performing mixture, followed by the asphalt mixtures with Paveflex140 and EE-2 polymers.

## Figures and Tables

**Table 1:** Properties of asphalt cement.

Properties	Unit	References	Values
High-temperature grade	°C	ASTM-D7175	64
Low-temperature grade	°C	ASTM-D6648	−22
G*/sinδ @ 64 °C	-	-	1.62
Penetration @ 25 °C	0.1 mm	ASTM-D0005	68
Softening Point	°C	ASTM-D0036	48
Flash Point	°C	ASTM-D1310	300
Penetration index	-	-	−0.99
Ductility	cm	ASTM-D0113	+100
Rotational viscosity @ 135 °C, cps	cp	ASTM-D4402	487
Rotational viscosity @ 165 °C, cps	cp	ASTM-D4402	150
Specific gravity	-	ASTM-D0070	1.025
Loss after RTFO	%	ASTM-D2872	0.07

**Table 2:** Aggregate gradation and MOT specification.

Sieve Opening (mm)	% Passing	Specification	
19.00	100.0		100
12.50	94.5	90	100
9.50	89.5		90
4.75	71.3		
2.36	49.1	28	58
1.18	30.3		
0.60	18.0		
0.30	9.0		
0.15	5.2		
0.075	3.2	2	10

**Table 3:** The limestone aggregate properties.

Property	Test Method	Value
Percentage loss by Los Angeles Abrasion Test, %	ASTM-C0131	21
Flat and Elongated Particles, %	ASTM-D4791	7
The Specific gravity of coarse aggregate	ASTM-C0127	2.585
Water absorption of coarse aggregate, %	ASTM-C0127	2.7
The Specific gravity of fine aggregate	ASTM-C0128	2.567
Water absorption of fine aggregate, %	ASTM0C0128	2.2

**Table 4:** Polymer’s properties.

Modifiers	Physical Form	Density (g/cm^3^)	Melting Point (°C)	Melt Flow Index (g/10 min)	Components
Lucolast7010	Pellet	0.924	95	3.9	Ethylene and Butyl Acrylate (EBA) with low crystallinity.
Anglomak2144	Pellet	0.930	96	3.5	Oxidized Polyethylene Homopolymer.
Paveflex140	Powder	-	212	-	Ethylene Vinyl Acetate Resins.
SBS KTR 401	Pellet	0.94	270	<1	Styrene Butadiene Styrene.
EE-2	Pellet	0.96	-	-	Medium-Density Oxidized Polyethylene.

**Table 5:** The rheological and physical properties of PMA.

Modifiers	Code	%	Penetration	Softening Point	G*/sinδ			m-Value			PG
					64 °C	70 °C	76 °C	−10 °C	−16 °C	−22 °C	
Lucolast7010	LU	3.6	36	59	5.69	2.68	1.37	0.366	0.315	0.285	76-16
Anglomak 2144	AM	3.2	34	60.8	7.51	3.39	1.68	0.312	0.279	-	76-10
Pavflex140	PF	5.0	34	59.2	6.25	2.99	1.49	0.320	0.290	-	76-10
SBS KTR 401	SBS	3.0	47	55	3.47	1.77	1.01	0.367	0.311	0.287	76-16
EE-2	EE	4.0	35	67	4.38	2.27	1.35	0.309	0.288	-	76-10

Note: G* = complex modulus; δ = phase angle; m-value = the tangent of the creep curve; PG = performance grade.

**Table 6:** Volumetric properties for different asphalt binder content.

Property	Values				MOT Specification
Binder Content, %	4.5	5.0	5.5	6.0	-
%Gmm @ N_ini_, %	83.1	85.5	87.4	88.4	≤89
%Gmm @ N_des_, %	92.2	95.2	97.3	98.0	96
Air Voids, %	7.8	4.8	2.7	2.0	4
VMA, %	14.9	14.1	13.3	13.3	≥14
VFA, %	47.6	65.6	79.4	85.0	65–75
Effective Binder Content (P_be_)	3.1	4.0	4.6	4.9	-
Dust Proportion (DP ratio)	0.99	0.77	0.68	0.64	0.6–1.2

**Table 7:** Volumetric properties for neat and PMA mixtures.

Property	Control	Anglomak	SBS	Lucolast	Pavflex	EE-2	Criteria
Gmm @ Nini, %	85.7	86.9	86.6	86.5	85.5	85.9	≤89
Gmm @ Ndes, %	95.8	96.6	96.1	96.2	95.4	96	96.0
Air Voids, %	4.2	3.4	3.9	3.8	4.6	4.0	3.0–5.0
VMA, %	13.7	13.0	13.2	13.1	13.8	13.9	14
VFA, %	68	73.3	70.4	70.8	66.7	71	65–75

**Table 8:** Effect sizes dynamic modulus at the temperature of −10 °C.

	NEAT	LU	AM	PF	SBS	EE
NEAT	-	4.96	6.04	9.01	9.52	2.66
LU	4.96	-	2.81	8.15	9.48	0.26
AM	6.04	2.81	-	2.66	3.15	1.62
PF	9.01	8.15	2.66	-	0.57	3.21
SBS	9.52	9.48	3.15	0.57	-	3.47
EE	2.66	0.26	1.62	3.21	3.47	-

**Table 9:** Effect sizes dynamic modulus at the temperature of 4.4 °C.

	NEAT	LU	AM	PF	SBS	EE
NEAT	-	1.11	7.09	3.59	99.74	2.16
LU	1.11	-	7.21	3.74	63.32	1.93
AM	7.09	7.21	-	1.75	6.47	6.30
PF	3.59	3.74	1.75	-	7.61	4.10
SBS	99.74	63.32	6.47	7.61	-	13.95
EE	2.16	1.93	6.30	4.10	13.95	-

**Table 10:** Effect sizes dynamic modulus at the temperature of 21.1 °C.

	NEAT	LU	AM	PF	SBS	EE
NEAT	-	5.00	7.64	10.38	5.75	0.83
LU	5.00	-	5.17	11.48	1.01	3.92
AM	7.64	5.17	-	1.62	4.95	6.92
PF	10.38	11.48	1.62	-	16.62	9.20
SBS	5.75	1.01	4.95	16.62	-	4.62
EE	0.83	3.92	6.92	9.20	4.62	-

**Table 11:** Effect sizes dynamic modulus at the temperature of 54.4 °C.

	NEAT	LU	AM	PF	SBS	EE
NEAT	-	6.58	11.80	8.83	2.76	4.09
LU	6.58	-	3.03	0.42	5.45	2.84
AM	11.80	3.03	-	2.96	10.43	6.55
PF	8.83	0.42	2.96	-	7.35	3.75
SBS	2.76	5.45	10.43	7.35	-	2.65
EE	4.09	2.84	6.55	3.75	2.65	-

**Table 12:** Flow number test data for different mixtures.

Asphalt Mixture	Fn		Failure	
	Cycles	Strain	Cycles	Strain
Control	25	19,683	66	52,377
Lucolast7010	182	23,652	432	50,184
Anglomk2144	120	18,982	336	50,292
Paveflex140	93	20,209	244	50,058
SBS KTR401	33	16,811	93	52,246
EE-2	46	21,338	114	51,111

**Table 13:** Effect sizes of Fn.

	NEAT	LU	AM	PF	SBS	EE
NEAT	-	7.54	8.29	17.86	2.22	5.09
LU	7.54	-	2.65	4.33	7.26	6.60
AM	8.29	2.65	-	2.49	7.97	6.67
PF	17.86	4.33	2.49	-	37.95	18.43
SBS	2.22	7.26	7.97	37.95	-	5.81
EE	5.09	6.60	6.67	18.43	5.81	-

**Table 14:** Indirect tensile strength for different mixtures.

Asphalt Mixture	Tensile Strength, kPa	
	Dry Condition	Wet Condition
Control	1027.5	843.6
Lucolast7010	990.8	859.3
Anglomk2144	1029.7	917.5
Paveflex140	1086.9	940.20
SBS KTR401	1139.6	1033.7
EE-2	957.8	861.6

**Table 15:** Effect sizes for *TSR*.

	NEAT	LU	AM	PF	SBS	EE
NEAT	-	1.09	3.28	1.97	2.95	3.42
LU	1.09	-	0.50	0.12	0.81	0.70
AM	3.28	0.50	-	1.83	0.71	0.60
PF	1.97	0.12	1.83	-	1.72	2.09
SBS	2.95	0.81	0.71	1.72	-	0.31
EE	3.42	0.70	0.60	2.09	0.31	-

**Table 16:** Asphalt mixture ranking.

Property	Mix Design					
	Control	Lucolast7010	Anglomk2144	Paveflex140	SBS KTR401	EE-2
ITS-Dry	4	5	3	2	1	6
ITS-Wet	6	5	3	2	1	4
*TSR*	6	4	3	5	1	2
Fn	6	1	2	3	5	4
E* at −10	1	3	4	6	5	2
E* at 4.4	3	2	5	4	6	1
E* at 21.1	6	4	1	2	3	5
E* at 54.4	6	3	1	2	5	4
HWT	6	4	2	3	5	1
Sum	44	31	24	29	32	29
Relative index	0.352	0.593	0.722	0.630	0.574	0.630
Overall Ranking	6	4	1	2	5	3

## Data Availability

All data used in this research can be provided upon request.

## References

[1] Costa L.M., Silva H.M.R.D., Oliveira J.R., Fernandes S.R. (2013). Incorporation of waste plastic in asphalt binders to improve their performance in the pavement. Int. J. Pavement Res. Technol..

[2] Gama D.A., Júnior J.M.R., Melo T., Rodrigues J.K.G. (2016). Rheological studies of asphalt modified with elastomeric polymer. Constr. Build. Mater..

[3] Mansourian A., Goahri A.R., Khosrowshahi F.K. (2019). Performance evaluation of asphalt binder modified with EVA/HDPE/nanoclay based on linear and non-linear viscoelastic behaviors. Constr. Build. Mater..

[4] Shafabakhsh G., Rajabi M., Sahaf A. (2019). The fatigue behavior of SBS/nanosilica composite modified asphalt binder and mixture. Constr. Build. Mater..

[5] Babalghaith A.M., Koting S., Sulong N.H.R., Karim M.R. (2019). Optimization of mixing time for polymer modified asphalt. Proceedings of the IOP Conference Series: Materials Science and Engineering, 10th Malaysian Road Conference & Exhibition.

[6] Yan K., You L., Wang D. (2019). High-Temperature Performance of Polymer-Modified Asphalt Mixes: Preliminary Evaluation of the Usefulness of Standard Technical Index in Polymer-Modified Asphalt. Polymers.

[7] Bansal S., Misra A.K., Bajpai P. (2017). Evaluation of modified bituminous concrete mix developed using rubber and plastic waste materials. Int. J. Sustain. Built Environ..

[8] Kebria D.Y., Moafimadani S., Goli Y. (2015). Laboratory investigation of the effect of crumb rubber on the characteristics and rheological behaviour of asphalt binder. Road Mater. Pavement Des..

[9] Milad A., Ahmeda A.G.F., Taib A.M., Rahmad S., Solla M., Yusoff N.I.M. (2020). A review of the feasibility of using crumb rubber derived from end-of-life tire as asphalt binder modifier. J. Rubber Res..

[10] Khan M.Z.H., Koting S., Katman H.Y.B., Ibrahim M.R., Babalghaith A.M., Asqool O. (2021). Performance of High Content Reclaimed Asphalt Pavement (RAP) in Asphaltic Mix with Crumb Rubber Modifier and Waste Engine Oil as Rejuvenator. Appl. Sci..

[11] Gawdzik B., Matynia T., Błażejowski K. (2020). The Use of De-Vulcanized Recycled Rubber in the Modification of Road Bitumen. Materials.

[12] Ameri M., Mansourian A., Sheikhmotevali A.H. (2013). Laboratory evaluation of ethylene vinyl acetate modified bitumens and mixtures based upon performance related parameters. Constr. Build. Mater..

[13] Köfteci S., Ahmedzade P., Kultayev B. (2014). Performance evaluation of bitumen modified by various types of waste plastics. Constr. Build. Mater..

[14] Ameri M., Yeganeh S., Valipour P.E. (2019). Experimental evaluation of fatigue resistance of asphalt mixtures containing waste elastomeric polymers. Constr. Build. Mater..

[15] Peng C., Guo C., You Z., Xu F., Ma W., You L., Li T., Zhou L., Huang S., Ma H. (2020). The Effect of Waste Engine Oil and Waste Polyethylene on UV Aging Resistance of Asphalt. Polymers.

[16] Anwar M., Shah S., Alhazmi H. (2021). Recycling and Utilization of Polymers for Road Construction Projects: An Application of the Circular Economy Concept. Polymers.

[17] Milad A., Ali A.S.B., Babalghaith A.M., Memon Z.A., Mashaan N.S., Arafa S. (2021). Utilisation of Waste-Based Geopolymer in Asphalt Pavement Modification and Construction; A Review. Sustainability.

[18] Jeffry S.N.A., Jaya R.P., Hassan N.A., Yaacob H., Mirza J., Drahman S.H. (2018). Effects of nanocharcoal coconut-shell ash on the physical and rheological properties of bitumen. Constr. Build. Mater..

[19] Ramadhansyah P., Irwan R.N., Idris A.M., Ezree A.M., Khatijah A.S., Norhidayah A., Haryati Y. (2019). Stability and voids properties of hot mix asphalt containing black rice husk ash. Proceedings of the IOP Conference Series: Earth and Environmental Science, National Colloquium on Wind and Earthquake Engineering.

[20] Rusbintardjo G., Hainin M.R., Yusoff N.I.M. (2013). Fundamental and rheological properties of oil palm fruit ash modified bitumen. Constr. Build. Mater..

[21] Saltan M., Terzi S., Karahancer S. (2017). Examination of hot mix asphalt and binder performance modified with nano silica. Constr. Build. Mater..

[22] Lu X., Isacsson U. (1997). Rheological characterization of styrene-butadiene-styrene copolymer modified bitumens. Constr. Build. Mater..

[23] Perezlepe A. (2003). Influence of the processing conditions on the rheological behaviour of polymer-modified bitumen ☆. Fuel.

[24] Topal A. (2010). Evaluation of the properties and microstructure of plastomeric polymer modified bitumens. Fuel Process. Technol..

[25] Bernier A., Zofka A., Yut I. (2012). Laboratory evaluation of rutting susceptibility of polymer-modified asphalt mixtures containing recycled pavements. Constr. Build. Mater..

[26] Modarres A. (2013). Investigating the toughness and fatigue behavior of conventional and SBS modified asphalt mixes. Constr. Build. Mater..

[27] Kök B.V., Çolak H. (2011). Laboratory comparison of the crumb-rubber and SBS modified bitumen and hot mix asphalt. Constr. Build. Mater..

[28] Lu X., Isacsson U., Ekblad J. (1998). Low-temperature properties of styrene–butadiene–styrene polymer modified bitumens. Constr. Build. Mater..

[29] Behnood A., Gharehveran M.M. (2019). Morphology, rheology, and physical properties of polymer-modified asphalt binders. Eur. Polyme. J..

[30] Vargas C., El Hanandeh A. (2021). Systematic literature review, meta-analysis and artificial neural network modelling of plastic waste addition to bitumen. J. Clean. Prod..

[31] Özdemir D.K., Topal A., Sengoz B. (2020). The influences of altering the mixing conditions on the properties of polymer modified bitumen: An overview. Uludağ Univ. J. Fac. Eng..

[32] Airey G.D. (2003). Rheological properties of styrene butadiene styrene polymer modified road bitumens⋆. Fuel.

[33] Giuliani F., Merusi F., Filippi S., Biondi D., Finocchiaro M.L., Polacco G. (2009). Effects of polymer modification on the fuel resistance of asphalt binders. Fuel.

[34] Sengoz B., Isikyakar G. (2008). Analysis of styrene-butadiene-styrene polymer modified bitumen using fluorescent microscopy and conventional test methods. J. Hazard. Mater..

[35] Sengoz B., Topal A., Isikyakar G. (2009). Morphology and image analysis of polymer modified bitumens. Constr. Build. Mater..

[36] Bulatović V.O., Rek V., Marković K.J. (2013). Rheological properties and stability of ethylene vinyl acetate polymer-modified bitumen. Polym. Eng. Sci..

[37] Babalghaith A.M., Alsoliman H.A., Al-Suhaibani A.S. (2016). Comparison of rheological properties for polymer modified asphalt produced in riyadh. Int. J. Civil Environ. Eng..

[38] Milad A.A., Ali A.S.B., Yusoff N.I.M. (2020). A Review of the Utilisation of Recycled Waste Material as an Alternative Modifier in Asphalt Mixtures. Civ. Eng. J..

[39] Wei J., Liu Z., Zhang Y. (2013). Rheological properties of amorphous poly alpha olefin (APAO) modified asphalt binders. Constr. Build. Mater..

[40] Wang H., You Z., Mills-Beale J., Hao P. (2012). Laboratory evaluation on high temperature viscosity and low temperature stiffness of asphalt binder with high percent scrap tire rubber. Constr. Build. Mater..

[41] Fernandes M.R.S., Forte M.M.C., Leite L.F.M. (2008). Rheological evaluation of polymer-modified asphalt binders. Mater. Res..

[42] Robbins M.M. (2009). An Investigation Into Dynamic Modulus of Hot-Mix Asphalt and its Contributing Factors, in Civil Engineering.

[43] Pareek A., Gupta T., Sharma R.K. (2012). Performance of Polymer Modified Bitumen for Flexible Pavements. Int. J. Struct. Civil Eng. Res..

[44] Li Q., Ni F., Li G., Wang H. (2013). Evaluation of the dynamic modulus for asphalt mixtures with varying volumetric properties. Int. J. Pavement Res. Technol..

[45] Zhu H., Sun L., Yang J., Chen Z., Gu W. (2011). Developing Master Curves and Predicting Dynamic Modulus of Polymer-Modified Asphalt Mixtures. J. Mater. Civ. Eng..

[46] Ping W., Xiao Y. Evaluation of SBS Polymer Binder Effect on Resilient Modulus Properties of Florida HMA Mixtures. Proceedings of the 24th OCTPA Annual Conference and NACGEAI International Symposium on Geo-Trans.

[47] Modarres A., Hamedi H. (2014). Effect of waste plastic bottles on the stiffness and fatigue properties of modified asphalt mixes. Mater. Des..

[48] Fakhri M., Hassani K., Ghanizadeh A.R. (2013). Impact of Loading Frequency on the Fatigue behavior of SBS Modified Asphalt Mixtures. Procedia Soc. Behav. Sci..

[49] Al-Abdul-Wahhab H.I. (2012). Effect of Modifiers and Additives on Fatigue Behavior of Asphalt Concrete Mixes in the Gulf. J. Pavement Res. Technol..

[50] Xu Q., Chen H., Prozzi J.A. (2010). Performance of fiber reinforced asphalt concrete under environmental temperature and water effects. Constr. Build. Mater..

[51] Arabani M., Mirabdolazimi S., Sasani A. (2010). The effect of waste tire thread mesh on the dynamic behaviour of asphalt mixtures. Constr. Build. Mater..

[52] Hamdou H.M., Ismael M.Q., Abed M.A. (2014). Effect of Polymers on Permanent Deformation of Flexible Pavement. J. Eng..

[53] Özen H. (2011). Rutting evaluation of hydrated lime and SBS modified asphalt mixtures for laboratory and field compacted samples. Constr. Build. Mater..

[54] Fontes L.P., Trichês G., Pais J.C., Pereira P.A. (2010). Evaluating permanent deformation in asphalt rubber mixtures. Constr. Build. Mater..

[55] Abed A.H., Bahia H.U. (2020). Enhancement of permanent deformation resistance of modified asphalt concrete mixtures with nano-high density polyethylene. Constr. Build. Mater..

[56] Ameli A., Babagoli R., Khabooshani M., AliAsgari R., Jalali F. (2020). Permanent deformation performance of binders and stone mastic asphalt mixtures modified by SBS/montmorillonite nanocomposite. Constr. Build. Mater..

[57] Al-Dubabe I.A. (1996). Polymer Modification of Arab Asphalt to Suit Gulf Countries Performance Requirments in Civil Engineering.

[58] Wahhab H.I.A., Balghunaim F.A. (1994). Asphalt Pavement Temperature Related to Arid Saudi Environment. J. Mater. Civ. Eng..

[59] Wahhab H.I.A.-A., Asi I.M., Al-Dubabe I.A., Ali M.F. (1997). Development of performance-based bitumen specifications for the Gulf countries. Constr. Build. Mater..

[60] General Directorate for Material and Research (2006). Hot Asphalt Mix Design System.

[61] Al-Dubabe I.A., Wahhab H.I.A.-A., Asi I.M., Ali M.F. (1998). Polymer Modification of Arab Asphalt. J. Mater. Civ. Eng..

[62] National Asphalt Pavement Association (2002). Designing and Constructing SMA Mixtures: State of the Practice.

[63] Dongré R., D’Angelo J., Copeland A. (2009). Refinement of Flow Number as Determined by Asphalt Mixture Performance Tester: Use in Routine Quality Control—Quality Assurance Practice. Transp. Res. Rec. J. Transp. Res. Board.

[64] Bonaquist R.F., Christensen D.W., Stump W. (2003). Simple Performance Tester for Superpave Mix Design: First-Article Development and Evaluation.

[65] Bower N., Wen H., Wu S., Willoughby K., Weston J., Devol J. (2015). Evaluation of the performance of warm mix asphalt in Washington state. Int. J. Pavement Eng..

[66] Tan G., Wang W., Cheng Y., Wang Y., Zhu Z. (2020). Master Curve Establishment and Complex Modulus Evaluation of SBS-Modified Asphalt Mixture Reinforced with Basalt Fiber Based on Generalized Sigmoidal Model. Polymers.

[67] Khattak M.J. (1999). Engineering Characteristics of Polymer Modified Asphalt Mixtures, in Department of Civil and Environmental Engineering.

[68] Alsoliman H.A. (2010). Engineering Characteristics of Local Polymer Modified Asphalt Mixtures. Ph.D. Thesis.

[69] Kumar P., Chandra S., Bose S. (2006). Strength characteristics of polymer modified mixes. Int. J. Pavement Eng..

[70] Babalghaith A.M., Alsolieman H.A., Al-Suhaibani A.S., Koting S. (2020). Master curve of dynamic modulus for modified asphalt mixtures. AIP Conference Proceedings.

